# Association of TLR-2 Gene Polymorphisms with the Risk of Periodontitis: A Meta-Analysis

**DOI:** 10.1155/2020/9353958

**Published:** 2020-08-06

**Authors:** Chao Shan, Abasijiang Aisaiti, Zhong Peng Wu, Ting Ting Wang, Jin Zhao

**Affiliations:** Department of Endodontics, First Affiliated Hospital of Xinjiang Medical University (Affiliated Stomatological Hospital), Urumqi 830054, China

## Abstract

**Background:**

Periodontitis is a kind of chronic infectious disease, affecting the health of billions of people. In recent years, a number of studies have shown that multiple immune gene polymorphisms are associated with the susceptibility to periodontitis, among which TLR-2 plays a critical role in periodontitis. But most of the studies reported TLR-2 gene polymorphism and susceptibility to periodontitis are not consistent. Therefore, we included all eligible studies in our study for further meta-analysis.

**Methods:**

We used electronic databases, including CNKI, PubMed, EMBASE, and Web of Science databases, and relevant research published through June, 2020. Selecting studies involved case-control trials. For all eligibility studies, odds ratios (ORs) and 95% confidence intervals (95% CI) are provided or can be calculated from the study data. The size of the combined effect was calculated using STATA 15.0.

**Results:**

Our meta-analysis included 14 articles representing 18 case-control studies with a total of 3873 cases and 3438 control subjects. Significant association was found between periodontitis and TLR-2 rs1898830 polymorphism under the allelic model (A allele vs. G allele: *p* = 0.014, OR = 1.208, 95% CI: 1.039-1.406), recessive model (GG vs. GA+AA: *p* = 0.028, OR = 0.755, 95% CI: 0.588-0.970), and codominant model (GG VS. AA: *p* = 0.014, OR = 0.681, 95% CI: 0.501-0.925). In subgroup analysis, TLR-2 rs5743708 polymorphism was associated with periodontitis risk in Asians under an allelic model (G allele vs. A allele: *p* = 0.017, OR = 12.064, 95% CI: 1.570-92.688), dominant model (GA+AA vs.GG: *p* = 0.016, OR = 0.08, 95% CI: 0.010-0.620), and codominant model (GA VS. GG: *p* = 0.016, OR = 1.026, 95% CI: 0.821-1.282).

**Conclusion:**

The TLR-2 rs1898830, rs5743708 polymorphism may be associated with susceptibility to periodontitis. In the future, genome-wide approaches and large-scale, multiethnic case-control trials are still needed.

## 1. Introduction

Periodontitis is a chronic infectious disease caused by the accumulation of bacteria such as P.g (Porphyromonas gingivalis) and A.a (Aggregatibacter actinomycetemcomitans), which leads to the destruction of the surrounding periodontal tissue, including deep pockets, loss of attachment, and loss of the alveolar bone [1, 2]. Moreover, it is the main cause of adult tooth loss, which requires huge expenditure for the prevention and treatment of periodontal disease every year [3, 4]. Currently, the idea that plaques and microorganism's well-characterized shift—the change from the gram-positive to the gram-negative was in the process of the change from periodontal health to periodontal disease—are the initial factors of periodontitis. P.g, Tannerella forsythia, and Treponema denticola are designated the “red-complex” periopathogens and have been studied extensively [5]. A large number of previous studies have confirmed that both periodontitis are caused by a complex combination of bacterial infection and genetic factors, with heritability estimates as high as 50% [6]. Unlike some monogenic diseases usually caused by a single gene, genetic susceptibility to periodontitis can be determined by hundreds of genes, while clinical phenotypes are determined by the interaction of environmental, genetic, and epigenetic factors [7]. However, the most known or assumed genetic risk loci for chronic periodontitis (CP) are based on candidate gene studies; these small effect genes have small to moderate effects and explain only a small fraction of disease heritability for periodontitis. Recently, some studies have focused on genome-wide approaches rather than candidate gene approaches, thus considering the possibility that multiple genes may be causal factors for periodontitis [8–10].

Toll-like receptors (TLRs) are kinds of pattern recognition receptors (PRRs), which can be specific to identify pathogenic microorganisms that are conservative antigen molecules in evolution, such as lipopolysaccharide (LPS), which are the main components of the outside membrane of gram-negative bacteria, lipoproteins, or other parts of bacterial or fungal cell wall components, causing the host production of inflammatory cytokines and chemokines such as IL-18 and IL-1*β* [11]. Thus, PRRs monitoring the invasion of pathogenic microorganisms effectively induce the immune response and regulate innate inflammation [12]. The role of TLR-2 in the pathophysiology of periodontitis has been widely discussed; elevated levels of TLR-2 were found in gingival inflammatory tissue [13]. In addition, knockout experiments in mice suggested that TLR2 identifies the key periodontal bacteria like P.g and A.a in periodontal infection [14]. In Scheres et al.'s study, P.g's LPS was used to stimulate human gingival fibroblasts in vitro, causing higher expression levels of TLR1, TLR2, and TLR7 [15].

Interestingly, although numerous studies have demonstrated a link between TLR-2 and periodontitis susceptibility, focusing on certain SNPs like rs5743708 and rs1898830, they showed no positive conclusion from their findings. This could be due to a small sample size and incomplete capture of genetic variation, or previous studies focused only one or several SNPs. Genetic association studies designed to examine the relationship between genetic variation and complex results must be treated with caution, even if the results are negative, as many factors may affect the results [16]. The meta-analysis is aimed at (1) overcoming the shortage of small sample research, (2) conducting a comprehensive analysis, (3) objectively evaluating statistical methods, and (4) comprehensively applying various effects to achieve the same goal. This approach leads to more reliable conclusions than a single study. In recent years, meta-analysis has been widely used in gene polymorphism research. Therefore, the meta-analysis and subgroup analysis further clarify the relationship between TLR2 polymorphism and periodontitis susceptibility in order to reconcile inconsistencies across individual studies.

## 2. Materials and Methods

### 2.1. Protocols and Eligibility Criteria

The meta-analysis and systematic review reported here are in accordance with the Preferred Reporting Items for the Systematic Review and Meta-Analyses (PRISMA) statement [17]. The literature search was limited to original studies performed in humans on the association of Toll-like receptor 2 SNPs with periodontitis risk.

### 2.2. Search Strategy

Data were collected from the CNKI (China National Knowledge Infrastructure), PubMed (PubMed is an interface for the Medline database), Embase, and Web of Science databases up to June, 2020 (last access on June, 2020). The search terms comprised the following 3 items and were combined using the Boolean operator AND: gene name (including “TLR-2”, “Toll-Like Receptor 2” or “TLR 2” or “tlr-2” or “tlr-2”). genetic polymorphisms (including “genetic variant”, “genetic “variation”, “polymorphism”, “mutation” or “mutan“), and disease (including “periodontal disease”, “periodontitis”, “chronic periodontitis”, “CP” or “PD”) to identify studies describing associations of genetic polymorphisms with periodontitis susceptibility. Relevant publications were also identified via searches supplemented by a literature review. Published articles in Chinese and English were retrieved. Moreover, hand searches for references cited in the published original and review articles were also performed.

### 2.3. Selection Criteria

Two authors identified studies eligible for further review by performing an initial screen of identified titles or abstracts. Articles considered for inclusion in the meta-analysis had to meet the following inclusion criteria: (a) studies used validated genotyping methods (such as PCR-RFLP or TaqMan) to measure the association of SNPs in TLR-2 genes with periodontitis risk; (b) a case-control cohort or nested case-control design; (c) the full text of the studies was available, and the data of the studies were not duplicated in another manuscript; (d) the case group of patients had a clinical diagnosis of periodontitis, including CP patients and aggressive periodontitis (AgP) patients, whereas the control group was the periodontal healthy population; and (e) when multiple document data were identical or overlapping, the most recently published paper was selected. Reviews, editorials, and case reports were excluded. Both reviewers fully agreed on the eligibility of the included articles in this first screening. Then, we performed a second screening based on full-text review. Studies such as cohort studies or nested case-control design analyses are included. According to the objective of this analysis, studies were excluded if they did not provide enough information on genotype frequency or did not report sufficient genotype distribution for the calculation of odds ratios (ORs) and its variance. Besides, studies were also excluded if the genotype distributions of control subjects were varied from Hardy-Weinberg equilibrium (HWE). Moreover, the case groups which suffered not only from periodontitis but also from other diseases were also excluded.

### 2.4. Data Extraction and Quality Assessment

We extracted detailed data on the first author's name, year of publication, region of the study, disease type, genotype, and smoking status. Furthermore, the evidence of HWE in controls was verified through the application of an online software (http://www.oege.org/software/hwe-mr-calc.shtml). *p* value less than 0.05 of HWE was considered to be significant. Data extracted was performed independently by two investigators, and discrepancies were reviewed by a third reviewer. The quality of the observational studies was assessed using the Newcastle-Ottawa scale, and they were examined independently by two researchers (http://wwwohrica/programs/clinical_epidemiology/oxfordasp). Briefly, a quality score (0-9) was generated according to a maximum of 1 star for each item on selection. And the quality of each study was assessed by using the following methodological components: (1) subject selection and (2) comparability of subject.

### 2.5. Heterogeneity

Between-study and between-subgroup heterogeneities were evaluated by calculating the *I*^2^ statistic and the Cochrane Q (*χ*^2^) statistic, with a *p* value of 0.10 set for significance of the test of heterogeneity. *I*^2^, directly calculated from the Q statistic, indicates the percentage of variability in effect estimates because of true heterogeneity, rather than sampling error. *I*^2^ ranges from 0% to 100%, with 0% indicating the absence of any heterogeneity. Although absolute numbers for *I*^2^ are not available, values < 50% are considered to have low heterogeneity, and the effect is thought to be fixed. Conversely, when *I*^2^ exceeds 50%, then heterogeneity is thought to exist and the effect is random.

### 2.6. Statistical Analysis

We performed a detailed meta-analysis to evaluate the association between TLR-2 SNPs and periodontitis susceptibility. We used five models, including the allele model, codominant models, dominant model, and recessive model, and subgroup analyses were performed based on the case type and racial descent. All data were analyzed using Stata version 15.0. Odds ratios (OR) and 95% CIs were calculated as the parameter of efficacy. *I*^2^ > 50% could suggest heterogeneity and suggest the random-effect model (DerSimonian-Laird) for the secondary endpoint. Otherwise, the fixed-effect model (Mantel-Haenszel) for the primary was used to calculate pooled ORs. Two-sided probability values of <0.05 were considered statistically significant. Potential publication bias of studies with different sample sizes was examined by visual inspection of funnel plots. This study is registered with PROSPERO, number CRD42020154093.

## 3. Results

### 3.1. Study Selection and Characteristics

**Figure 1:**
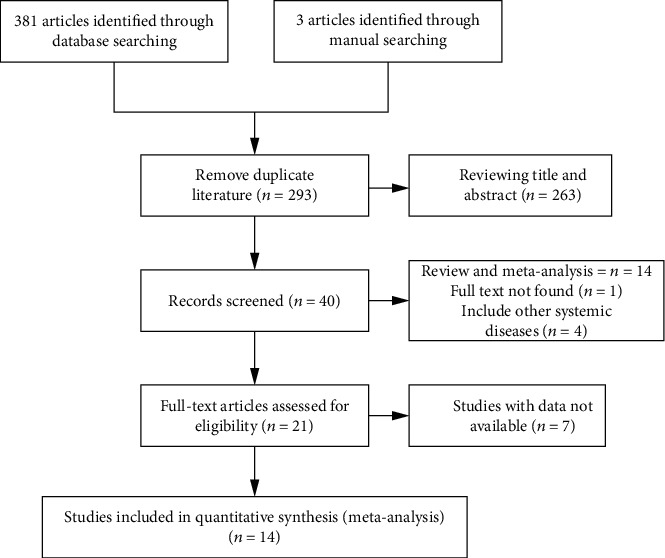
Flow diagram of the systematic review process.

Our search identified a total of 381 potentially relevant studies (Figure 1). No previous meta-analysis on this issue was identified. After removal of duplicate publications, 293 articles remained. By reviewing the title and abstract, 233 articles were excluded. The full text of the remaining 60 articles was reviewed in detail, and 46 of these were subsequently excluded. Therefore, fourteen articles representing eighteen case-control studies involving 3873 patients and 3438 control subjects were included in our meta-analysis [18–31]. Comprising CP and/or AgP were comprehensively assessed against the inclusion criteria. Two of the eligible articles that were written in Chinese other than English were retrieved and translated in order to avoid publication bias. We described the main features of the eligible papers. A total of 6 gene polymorphism studies were included in this meta-analysis (rs1898830, rs5743704, rs5743708, rs3804100, rs13150331, and rs12191786), and other SNPs of TLR-2 such as rs7696323 and rs5743709 were reported only in one study. About the ethnic issue, there were ten studies taking the Caucasian as the research object, while eight studies were contraposing the Asian population. Five studies were excluded because they were not in accordance with HWE. Besides, one more study was excluded due to insufficient data availability for calculating ORs and their variance [31]. And the characteristics and quality assessment of all included studies are summarized in Figure 1 and Table 1.

### 3.2. TLR-2 rs1898830 Polymorphism

**Figure 2:**
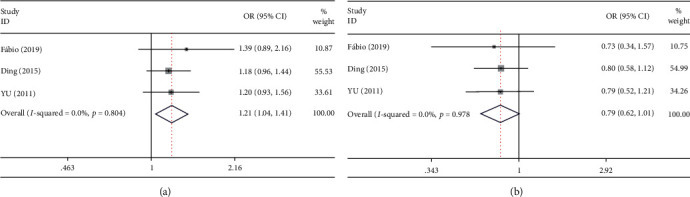
Forest plot of published case-control association studies of rs1898830 (A>G) in overall analysis fulfilling HWE. (a) Allele comparison (A allele vs. G allele). (b) Recessive model (GG vs. GA+AA).

The pooled ORs from the overall study with 2849 periodontitis patients and 2922 controls found a significant association between rs1898830 and chronic periodontitis risk under the allelic model (A allele vs. G allele: *p* = 0.014, OR = 1.208, 95% CI: 1.039-1.406), recessive model (GG vs. GA+AA: *p* = 0.028, OR = 0.755, 95% CI: 0.588-0.970), and codominant model (GG VS. AA: *p* = 0.014, OR = 0.681, 95% CI: 0.501-0.925). In the subgroup analysis, the association was found only in Asian populations under the allelic model (A allele vs. G allele: *p* = 0.037, OR = 1.186, 95% CI: 1.010-1.394) and codominant model (GG VS. AA: *p* = 0.035, OR = 0.704, 95% CI: 0.508-0.975) [18, 19, 25, 29] .The heterogeneity test for the pooled data sets was not significant (*I*^2^ = 0) for the TLR-2 rs1898830 polymorphism in five gene models, indicating the robustness of the meta-analysis for chronic periodontitis. Although a limited number of studies (*n* = 2) have been included for the rs1898830 polymorphism, it suggested that rs1898830 (A>G) may be associated with periodontitis risk in Asians (Figure 2 and Table 2).

### 3.3. TLR-2 rs3804100 Polymorphism

**Figure 3:**
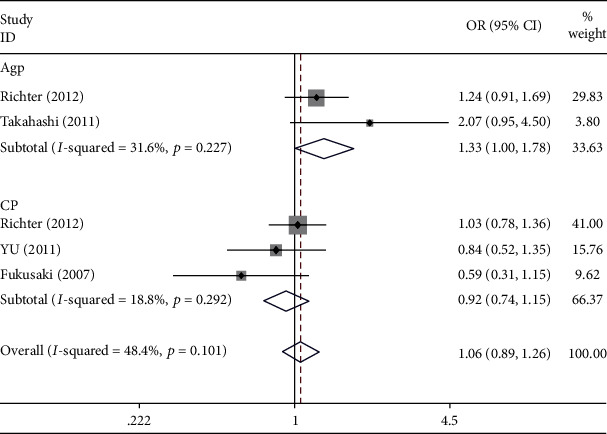
Forest plot of published case-control association studies of rs3804100 (T>C) in disease subgroup analysis fulfilling HWE. Codominant model (TC VS. TT).

Seven studies with 2857 cases and 2452 controls did not report an association between periodontitis and TLR-2 rs3804100 polymorphism in five gene models (*p* = 0.269, OR = 0.922, 95% CI: 0.797-1.065). No association was found between the ethnic subgroup analysis and the disease type subgroup analysis. Subgroup analysis by disease type found that the marginal significance was in the codominant model of TC VS. TT (*p* = 0.051, OR = 1.332, 95% CI: 0.999-1.777); statistical heterogeneity varied from absent to moderate especially among Asians and AgP subgroup analysis (*I*^2^ = 66.9% and *I*^2^ = 55.3%, respectively). Moreover, the genotype distributions of all the included studies were in accordance with HWE except for the study of Ding et al. (Figure 3 and Table 3).

### 3.4. TLR-2 rs5734704 Polymorphism

**Figure 4:**
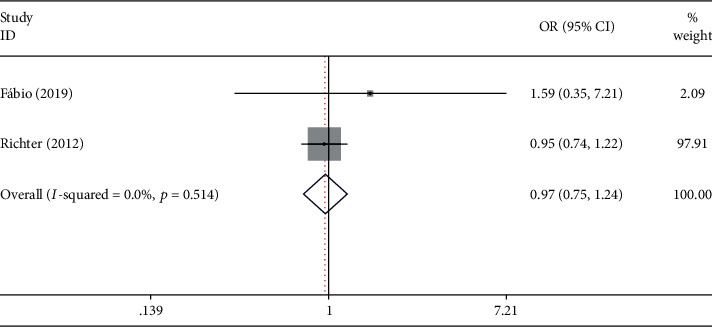
Forest plot of published case-control association studies of rs5734704 (C>A) in overall analysis fulfilling HWE. Allele comparison model (A allele vs. G allele).

Three studies derived from Caucasian ethnic involved 1596 cases and 1866 controls report the gene and showed that TLR-2 rs5734704 polymorphism might not contribute to periodontitis risk under all comparison models (*p* = 0.783, OR = 0.966, 95% CI: 0.755-1.237) either in Caucasian or disease study (*p* = 0.748, OR = 1.053, 95% CI: 0.770-1.439) (Figure 4 and Table 4).

### 3.5. TLR-2 rs5743708 Polymorphism

**Figure 5:**
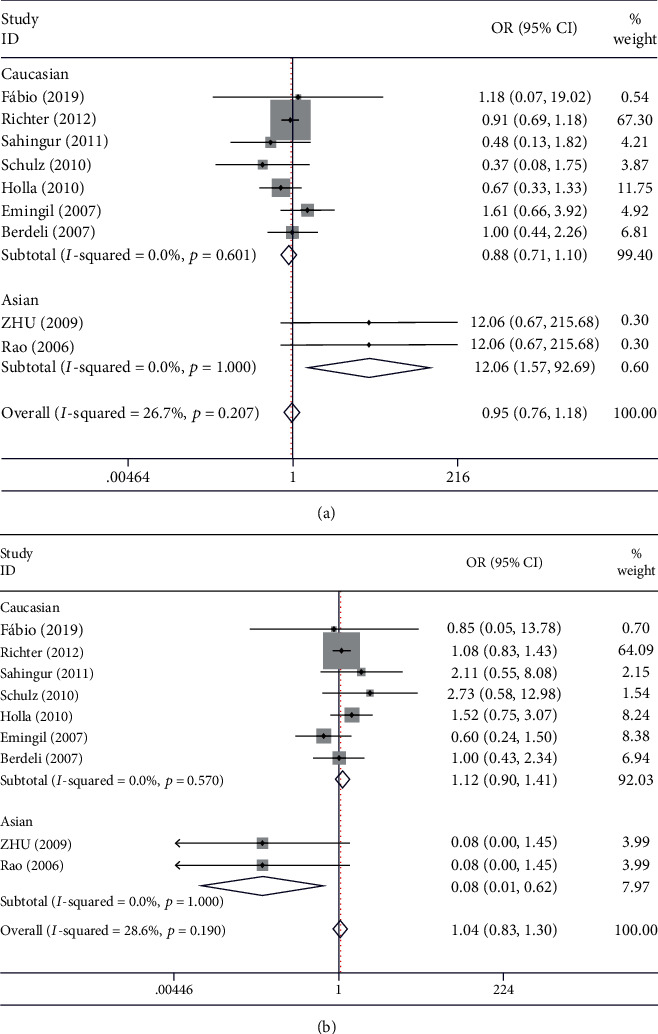
Forest plot of published case-control association studies of rs5743708 (G>A) in ethnic subgroup analysis fulfilling HWE. (a) allele comparison model (G allele vs. A allele) (b) dominant model (GA+AA vs.GG).

Thirteen studies with a total of 2361 cases and 2722 controls found no association between rs5743708 and periodontitis risk under the five gene models (*p* = 0.633, OR = 0.949, 95% CI: 0.765-1.177). Ethnic subgroup analyses showed that rs5743708 was associated with periodontitis risk in Asians under the allelic model (G allele vs. A allele: *p* = 0.017, OR = 12.064, 95% CI: 1.570-92.688), dominant model (GA+AA vs.GG: *p* = 0.016, OR = 0.08, 95% CI: 0.010-0.620), and codominant model (GA VS. GG: *p* = 0.016, OR = 0.080, 95% CI: 0.821-1.282). Stratification by disease type indicated no association between the TLR-2 rs5743708 polymorphism and periodontitis in CP and AgP. The overall genotyping distribution in all studies was in HWE equilibrium [18–21, 23, 24, 27–30] (Figure 5 and Table 5).

### 3.6. TLR-2 rs13150331 Polymorphism

Only two studies research Asian populations and aggressive periodontitis about TLR-2 rs13150331 polymorphism and failed to find a significant association of this variant with the risk of AgP in Asian under all comparison models [25, 29] (*p* = 0.161, OR = 1.121, 95% CI: 0.955-1.316) (Table 6).

### 3.7. Other TLR-2 Polymorphism

One SNP, namely, rs12191786, has been reported in two studies, but both of them deviated from the HWE equilibrium; therefore, we excluded the SNPs from our meta-analysis [28, 30](data not shown). And other SNPs rs7696323 in the study by Takahashi et al. have been investigated [26], and the association was found between the rs7696323 polymorphism and periodontitis susceptibility (OR = 0.48, *p* = 0.038) in Asians. In Fukusaki et al.'s study, they reported three point mutations in the 5′-untranslated region and one synonymous mutation in the coding region of TLR2 that were identified at base-pair positions -183, -148, -146, and +2343 (corresponding to rs5743709), rs1816702, rs11938228, rs3804099 [22], and rs7656411 were reported in Richer et al.'s study [19], but they failed to find a significant association of this variant with the risk of CP in Asian populations.

### 3.8. Publication Bias Analysis

**Figure 6:**
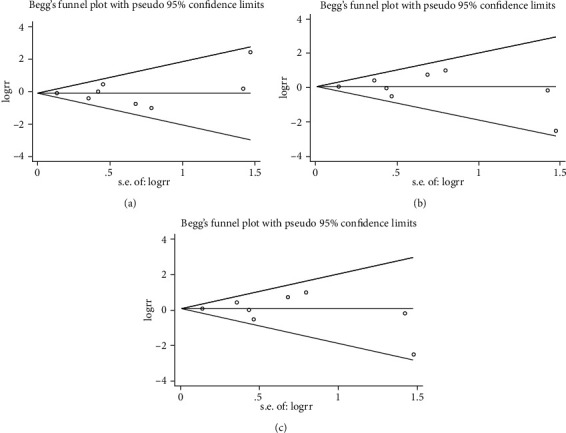
Begg's funnel plot of the TLR-2 rs5743708 (G>A) polymorphism and CP risk in different contrast models: (a) allele comparison model (G allele vs. A allele); (b) codominant model (GA vs. GG); and (c) dominant genetic model (AA+GA vs. GG).

The Begg-Mazumdar test and the modified Egger test for funnel plot asymmetry for all study groups did not indicate any evidence of publication bias for TLR-2 [32, 33] (Figure 6).

## 4. Discussion

This is the first meta-analysis which comprehensively performed to investigate the relationship between TLR-2 polymorphism and the risk of CP/AgP. We combined published data to assess the genetic association between the most commonly studied polymorphisms in the TLR-2 gene, including 18 studies with 3873 patients. The results showed that the rs1898830 (A>G) was at a decreased risk of chronic periodontitis in Asians under the allelic model (A VS. G) and codominant model (GG VS. AA). Four papers studying rs1898830 showed that the polymorphism of this gene was not significantly correlated with the periodontitis susceptibility [18, 19, 25, 29], which was inconsistent with the results of our meta-analysis. In Richer et al., the gene distribution of the control group in the study of this locus of gene rs1898830 did not conform to the HWE, so we did not include Richer data in the analysis of this gene polymorphism. On the other hand, these studies were largely unable to draw definitive conclusions from their findings, even if their results were negative. Therefore, it is reasonable to think that the allele mutation of this rs1898830 (A>G) may be a protective factor for CP.

There are more than 20 single nucleotide polymorphism (SNP) sites for the TLR2 gene included in the Hapmap database. Exon 3 is the largest exon in TLR2 and contains several synonymous and nonsynonymous SNPS, as shown in the NCBI (NCBI build GRCh37).We identified four known SNPs: two common variants (rs574304, rs5743708 (Arg753Gln)) and two rare variants (rs3804099, rs3804100), a missense mutation in the third exon of the TLR2 gene, which has been the most internationally reported to be associated with periodontitis. In total, ten publications have examined the role of TLR-2 rs5743708 genetic variation in periodontitis, suggesting that rs5743708 may not be related to the periodontal disease, which is consistent in previous papers [34, 35]. While ethnic subgroup analyses demonstrated that TLR-2 rs5743708 (G>A) was associated with Asian population under the allelic model (G allele vs. A allele) and dominant model (GA+AA vs.GG). This may be due to racial differences in genetic background. However, most previous studies have found that the occurrence of mutant genotypes/alleles is very rare. For example, Zhu et al. found only 6% heterozygotes in the control population in their study [28], so the study may be underpowered in this respect. There was no significant association between TLR-2 rs3804100 (T>C) among the overall and subgroup analysis. We found the marginal significance only in the codominant model on subgroup analysis in AgP. Among five studies related to the TLR-2 rs3804100 (T>C) and periodontitis, there was only one study conducted by Takahashi et al. which suggested that the rs3804100 (T>C) gene polymorphism in Asian subjects was associated with AgP [26]. Only two articles reported an association between this site and AgP in Asians. We could not conclude the significant result due to the small sample size. Additionally, no association was identified between the other TLR-2 polymorphism and periodontitis risk.

No significant association was observed for other sites mentioned in the papers between genetic TLR-2 polymorphisms of rs13150331 (A>G), rs12191786 (C>T), rs1816702 (C>T), rs11938228 (C>A), rs3804099 (T>C), rs7656411 (T>G), and base-pair positions -183 (A>G), -148 (C>T), -146 (T>G), and +2343 (G>A) (corresponding to rs5743709), except for one study, which reported decreased risk of rs7696323 polymorphism (C>T) in aggressive periodontitis in Asians [26]. As for rs12191786, three studies failed to identify the reported mutations rs12191786 in TLR-2. Fukusaki et al. performed direct sequencing of TLR2 and identified five base-pair positions -183, -148, -146, +1350 (corresponding to rs3804100) and +2343 (corresponding to rs5743709). Based on the associations observed in our meta-analysis, we concluded that most of these SNPs are only reported in one study and the mutant allele frequency between case and control was too small to have further analysis, because of the small sample size and the low frequency of alleles. Thus, the conclusions of the association between these SNPs and periodontitis are questionable.

In recent years, increasingly more studies on TLR single nucleotide polymorphisms (SNPs) have been reported. The main consideration factor is that TLR SNPs may reduce the response efficiency of TLR ligands, thus affecting the susceptibility to infectious diseases [36, 37]. Aggressive periodontitis and chronic periodontitis are complex diseases with multifactor etiology, genotyping a single genetic variation that often fails to draw conclusions about whether the genes in question are related to the disease. So, we identified several SNPs which had directly been investigated for its potential association with periodontitis in previous studies. Recently, some studies have shifted the focus to genome-wide approaches rather than candidate gene approaches, thereby considering the possibility that multiple genes may be causal factors in periodontitis. Schaefer et al. performed a genome-wide association study in two independent phases and found that the GLT6D1 gene was not significantly expressed in healthy tissues and gingival inflammatory tissues [38]. Thus, periodontitis appears to be associated with several sites, each of which has a relatively small effect. Only a few studies have investigated the cooccurrence of multiple polymorphisms, although more research may be forthcoming.

So far, a number of studies have evaluated the association between PRRs, SNPs, and periodontitis, including CD14, TLR2, TLR4, and MBL2. However, most of the studies on gene polymorphism of TLR-2 have considered that there is no obvious correlation between TLR-2 polymorphism and periodontitis. Therefore, the meta-analysis on the relationship between TLR-2 gene polymorphism and periodontitis susceptibility has not been reported alone, while the meta-analysis on TLR-4 and CD14 has been published; the conclusions are still inconsistent [34, 35, 39, 40]. Our meta-analysis was the first to independently analyze the relationship between TLR-2 and periodontitis, including 18 studies and six common SNPs and some unusual SNPs. We also found that the rs1898830 (A>G) may be a protective factor for periodontitis susceptibility, which has not been found in previous studies. In particualr, the rest of our results are consistent with previous studies. Although unlikely, rare disease-related variants may exist in these exons, which were not identified in our study. Currently, the availability of genome-wide arrays allows a wide range of genes to be scanned and identified in relation to disease processes. Although these methods were not used in these included studies and only a limited number of genes were studied, our results provide promising evidence for designing future studies to increase the sample size to determine genetic susceptibility to periodontitis in PRRs.

Our study has some limitations. First, the included studies varied in several ways, including age, gender, and smoking status. Only six articles cited smoking habits as a confounding factor. And we did not provide subgroup analysis for smoke status. Periodontitis is a multifactorial disease, and due to the lack of appropriate proportions of eligible studies, it is not possible to make detailed assessments of potential interactions, as gene-gene, gene-environment, or even different polymorphism loci of the same gene may regulate the risk of periodontitis. Second, due to the extremely low allele frequency of some SNPs such as rs5743704 (C>A), rs5743708 (G>A), and rs12191786(C>T), there is often no homozygous mutation or even no heterozygosity. For these SNPs, the calculation of some gene models (dominant model and a recessive model) cannot be carried out, which probably will affect our analysis results. In our meta-analysis, many individual studies seem to be underpowered. A sample size of 500-2000 case-control pairs is required to detect moderate genetic effect size [41]. And a large number of potential genetic risk factors and a relatively low frequency of polymorphisms may result in initial observations not being confirmed by subsequent studies. Although we have studied only a few SNPs of one gene, our results provide positive evidence for designing future studies to increase the sample size to determine genetic susceptibility to periodontitis in TLR.

## 5. Conclusions

In summary, this meta-analysis of published data shows that a protect correlation between the rs1898830 (A>G) and rs5743708 (G>A) was associated with periodontitis risk in Asians. Limited by the number of included samples and SNP sites in the current association study, the above conclusions need to be further confirmed by the large sample, multiethnics, and multi-SNP-sites studies in future. We consider that the genetic information of TLR2 site has a certain reference value for the diagnosis, prevention, and treatment of chronic periodontitis. If this genetic information is available, it may provide preventive strategies and therapeutic interventions for patients with high susceptibility.

## Figures and Tables

**Table 1:** Main characteristics of included studies.

References	Country	Ethnicity	Disease type	Genotype method	Polymorphism	Case/control (*n*)				Smoke status	HWE in controls	NOS
						11	12	22	Total			
Fábio (2019)	Denmark	Caucasian	CP	PCR–RFLP	rs1898830 (A>G) rs5743704 (C>A) rs5743708 (G>A)	21/14 83/69 85/72	45/34 3/4 1/1	20/25 0/0 0/0	86/73 86/73 86/73	NR	0.68 0.81 0.95	7
Ding (2015)	China	Asian	CP	PCR–RFLP	rs1898830 rs13150331 (A>G) rs3804100 (T>C)	190/62 177/64 221/79	339/131 350/129 245/108	136/62 141/64 176/63	665/255 668/257 642/250	Mixed	0.66 0.95 0.036	7
Richter (2012)	Germany	Caucasian	CP/AgP	TaqMan	rs1898830 rs3804100 rs5743704 rs5743708	667/1487 1300/1571 1394/1662 1343/1653	634/781 197/213 112/126 101/117	177/188 5/5 4/5 3/1	1478/2456 1502/1789 1510/1793 1447/1771	NR	0.001 0.43 0.12 0.46	7
YU (2011)	China	Asian	CP	TaqMan	rs13150331 rs1898830 rs3804100	167/36 181/34 157/33	328/68 315/70 223/56	128/36 124/34 203/40	623/140 620/138 583/129	Mixed	0.73 0.86 0.14	6
Takahashi (2011)	Japanese	Asian	AgP	PCR–RFLP	rs3804100	12/97	20/78	6/15	38/190	NR	0.90	7
Sahingur 2011	USA	Caucasian	CP	TaqMan	rs5743708	105/74	9/3	0/0	114/77	Mixed	0.86	7
Schulz 2010	Germany	Caucasian	AgP/CP	PCR–RFLP	rs5743708	130/79	9/2	0/0	139/81	NR	0.91	7
Holla 2010	Czech	Caucasian	CP	PCR–RFLP	rs5743708	203/244	19/15	0/0	222/259	Mixed	0.63	8
ZHU 2008	China	Asian	AgP/CP	PCR–RFLP	rs12191786 (C>T) rs5743708	0/0 90/94	90/100 0/6	0/0 0/0	90/100 90/100	NR	0.001 0.75	7
Fukusaki 2007	Japanese	Asian	CP	PCR–RFLP	rs3804100	66/56	21/30	5/8	92/94	NR	0.18	7
Emingil (2007)	Turkey	Caucasian	AgP	PCR–RFLP	rs5743708	83/136	7/19	0/0	90/155	Mixed	0.41	8
Berdeli (2007)	Turkey	Caucasian	CP	PCR–RFLP	rs5743708	72/92	11/14	0/0	83/106	Mixed	0.47	8
Rao (2006)	China	Asian	AgP/CP	PCR–RFLP	rs12191786 rs5743708	0/0 90/94	90/100 0/6	0/0 0/0	188/185 90/100	NR	0.001 0.76	6
Folwaczny (2004)	Germany	Caucasian	CP	PCR–RFLP	rs5743708 rs12191786	Only allele number available				NR	Unknown	5

TLR-2: toll-like receptor-2; CP: chronic periodontitis; AgP: aggressive periodontitis; 1: wild; 2: mutant; HWE: Hardy–Weinberg equilibrium; (A value less than 0.05 of HWE was considered significant); Smoke status: NR: not reported; NOS: Newcastle-Ottawa scale.

**Table 2:** Main outcomes of the analyses on periodontitis and TLR-2 rs1898830 (A>G) polymorphism.

Model	Subgroup	No. of studies	Case/control	OR	95% CI	*I* ^2^	Pooling model	*p* value
A vs. G	Overall (fulfill HWE)	3	1371/466	1.208	1.039-1.406	0	Fixed	0.014
	Caucasian	1	86/73	1.387	0.890-2.162	0	Fixed	0.149
	Asian	2	1285/393	1.186	1.010-1.394	0	Fixed	0.037
	CP	3	1371/466	1.208	1.039-1.406	0	Fixed	0.014
AG+GG vs. AA	Overall (fulfill HWE)	3	1371/466	0.792	0.619-1.015	0	Fixed	0.065
	Caucasian	1	86/73	0.734	0.343-1.575	0	Fixed	0.428
	Asian	2	1285/393	0.799	0.615-1.038	0	Fixed	0.093
	CP	3	1371/466	0.792	0.619-1.015	0	Fixed	0.065
GG vs. AG+AA	Overall (fulfill HWE)	3	1371/466	0.755	0.588-0.970	0	Fixed	0.028
	Caucasian	1	86/73	0.582	0.290-1.167	0	Fixed	0.127
	Asian	2	1285/393	0.787	0.601-1.029	0	Fixed	0.080
	CP	3	1371/466	0.755	0.588-0.970	0	Fixed	0.028
AG vs. AA	Overall (fulfill HWE)	3	1371/466	0.849	0.653-1.102	0	Fixed	0.218
	Caucasian	1	86/73	0.882	0.393-1.983	0	Fixed	0.762
	Asian	2	1285/393	0.845	0.641-1.113	0	Fixed	0.231
	CP	3	1371/466	0.849	0.653-1.102	0	Fixed	0.218
GG vs. AA	Overall (fulfill HWE)	3	1371/466	0.681	0.501-0.925	0	Fixed	0.014
	Caucasian	1	86/73	0.533	0.218-1.307	0	Fixed	0.169
	Asian	2	1285/393	0.704	0.508-0.975	0	Fixed	0.035
	CP	3	1371/466	0.681	0.501-0.925	0	Fixed	0.014

**Table 3:** Main outcomes of the analyses on periodontitis and TLR-2 rs3804100 (T>C) polymorphism.

Model	Subgroup	No. of studies	Case/control	OR	95% CI	*I* ^2^	Pooling model	*p*
T vs. C	Overall (fulfill HWE)	4	2215/2202	0.922	0.797-1.065	65.6%	Random	0.269
	Caucasian	1	1502/1789	0.898	0.738-1.092	0	Fixed	0.282
	Asian	3	713/413	0.951	0.766-1.179	76.7%	Random	0.645
	CP	3	1580/1302	1.012	0.849-1.207	43.4%	Fixed	0.893
	AgP	2	635/900	0.713	0.474-1.072	51.5%	Random	0.104
TC+CC vs. TT	Overall (fulfill HWE)	4	2215/2202	1.047	0.718-1.528	63.9%	Random	0.812
	Caucasian	1	1502/1789	1.120	0.912-1.375	0	Fixed	0.280
	Asian	3	713/413	1.033	0.530-2.014	74.1%	Random	0.924
	CP	3	1580/1302	0.942	0.758-1.170	31.8%	Fixed	0.589
	AgP	2	635/900	1.511	0.854-2.672	55.3%	Random	0.156
CC vs. TC+TT	Overall (fulfill HWE)	4	2215/2202	1.188	0.839-1.681	0	Fixed	0.332
	Caucasian	1	1502/1789	1.192	0.344-4.124	0	Fixed	0.782
	Asian	3	713/413	1.187	0.827-1.705	23.3%	Fixed	0.352
	CP	3	1580/1302	1.129	0.778-1.637	0	Fixed	0.523
	AgP	2	635/900	1.706	0.667-4.363	0	Fixed	0.265
TC vs. TT	Overall (fulfill HWE)	4	2215/2202	1.012	0.700-1.463	57.2%	Random	0.948
	Caucasian	1	1502/1789	1.118	0.909-1.375	0	Fixed	0.293
	Asian	3	713/413	0.918	0.653-1.290	66.9%	Random	0.621
	CP	3	1580/1302	0.921	0.735-1.154	18.8%	Fixed	0.474
	AgP	2	635/900	1.332	0.999-1.777	31.6%	Fixed	0.051^∗^
CC vs. TT	Overall (fulfill HWE)	4	2215/2202	1.122	0.749-1.681	40.6%	Fixed	0.577
	Caucasian	1	1502/1789	1.208	0.349-4.183	0	Fixed	0.765
	Asian	3	713/413	1.112	0.725-1.706	60.3%	Random	0.626
	CP	3	1580/1302	0.997	0.641-1.550	0	Fixed	0.990
	AgP	2	635/900	2.203	0.806-6.023	35.1%	Fixed	0.124

**Table 4:** Main outcomes of the analyses on periodontitis and TLR-2 rs5743704 (C>A) polymorphism.

Model	Subgroup	No. of studies	Case/control	OR	95% CI	*I* ^2^	Pooling model	*p*
C vs. A	Overall (fulfill HWE)	2	1596/1866	0.966	0.755-1.237	0	Fixed	0.783
	Caucasian	2	1596/1866	0.966	0.755-1.237	0	Fixed	0.783
	CP	2	998/1156	1.053	0.770-1.439	0	Fixed	0.748
	AgP	1	598/710	0.837	0.559-1.253	0	Fixed	0.386
CA+AA vs. CC	Overall (fulfill HWE)	2	1596/1866	1.040	0.805-1.344	0	Fixed	0.764
	Caucasian	2	1596/1866	1.040	0.805-1.344	0	Fixed	0.764
	CP	2	998/1156	0.954	0.690-1.319	0	Fixed	0.776
	AgP	1	598/710	1.203	0.791-1.831	0	Fixed	0.388
AA vs. CA+CC	Overall (fulfill HWE)	2	1596/1866	0.950	0.255-3.543	0	Fixed	0.939
	Caucasian	2	1596/1866	0.950	0.255-3.543	0	Fixed	0.939
	CP	2	998/1156	0.791	0.132-4.475	0	Fixed	0.798
	AgP	1	598/710	1.188	0.167-8.459	0	Fixed	0.863
CA vs. CC	Overall (fulfill HWE)	2	1596/1866	1.043	0.804-1.354	0	Fixed	0.749
	Caucasian	2	1596/1866	1.043	0.804-1.354	0	Fixed	0.749
	CP	2	998/1156	0.960	0.691-1.333	0	Fixed	0.806
	AgP	1	598/710	1.203	0.784-1.847	0	Fixed	0.397
AA vs. CC	Overall (fulfill HWE)	2	1596/1866	0.954	0.256-3.559	0	Fixed	0.944
	Caucasian	2	1596/1866	0.954	0.256-3.559	0	Fixed	0.944
	CP	2	998/1156	0.790	0.132-4.739	0	Fixed	0.797
	AgP	1	598/710	1.203	0.169-8.570	0	Fixed	0.853

**Table 5:** Main outcomes of the analyses on periodontitis and TLR-2 rs5743708 (G>A) polymorphism.

Model	Subgroup	No. of studies	Case/control	OR	95% CI	*I* ^2^	Pooling model	*p*
G vs. A	Overall (fulfill HWE)	9	2361/2722	0.949	0.765-1.177	26.7%	Fixed	0.633
	Caucasian	7	2181/2522	0.881	0.707-1.099	0	Fixed	0.262
	Asian	2	180/200	12.064	1.570-92.688	0	Fixed	0.017^∗^
	CP	8	1520/1823	0.845	0.646-1.104	0	Fixed	0.216
	AgP	5	841/1080	1.149	0.805-1.640	17.7%	Fixed	0.444
GA+AA vs. GG	Overall (fulfill HWE)	9	2361/2722	1.041	0.834-1.298	28.6%	Fixed	0.724
	Caucasian	7	2181/2522	1.124	0.897-1.409	0	Fixed	0.311
	Asian	2	180/200	0.080	0.010-0.620	0	Fixed	0.016^∗^
	CP	8	1520/1823	1.179	0.896-1.551	0	Fixed	0.241
	AgP	5	841/1080	0.849	0.590-1.221	18.8%	Fixed	0.377
AA vs. GA+GG	Overall (fulfill HWE)	9	2361/2722	3.677	0.382-35.389	0	Fixed	0.260
	Caucasian	7	2181/2522	3.677	0.382-35.389	0	Fixed	0.260
	Asian	2	180/200	—	—	—	Fixed	—
	CP	8	1520/1823	2.529	0.229-27.935	0	Fixed	0.449
	AgP	5	841/1080	2.865	0.422-19.464	0	Fixed	0.442
GA vs. GG	Overall (fulfill HWE)	9	2361/2722	1.026	0.821-1.282	0	Fixed	0.820
	Caucasian	7	2181/2522	1.109	0.883-1.392	0	Fixed	0.374
	Asian	2	180/200	0.080	0.821-1.282	0	Fixed	0.016^∗^
	CP	8	1520/1823	1.166	0.885-1.538	0	Fixed	0.275
	AgP	5	841/1080	0.833	0.578-1.201	17.5%	Fixed	0.328
AA vs. GG	Overall (fulfill HWE)	9	2361/2722	3.692	0.384-35.538	0	Fixed	0.258
	Caucasian	7	2181/2522	3.692	0.384-35.538	0	Fixed	0.258
	Asian	2	180/200	—	—	—	Fixed	—
	CP	8	1520/1823	2.555	0.231-28.229	0	Fixed	0.444
	AgP	5	841/1080	2.878	0.424-19.560	0	Fixed	0.444

**Table 6:** Main outcomes of the analyses on periodontitis and TLR-2 rs13150331 polymorphism.

Model	Subgroup	No. of studies	Case/control	OR	95% CI	*I* ^2^	Pooling model	*p*
A vs.G	Overall (fulfill HWE)	2	1291/397	1.121	0.955-1.316	0	Fixed	0.161
	Asian	2	1291/397	1.121	0.955-1.316	0	Fixed	0.161
	CP	2	1291/397	1.121	0.955-1.316	0	Fixed	0.161
AG+GG vs. AA	Overall (fulfill HWE)	2	1291/397	0.930	0.717-1.205	0	Fixed	0.581
	Asian	2	1291/397	0.930	0.717-1.205	0	Fixed	0.581
	CP	2	1291/397	0.930	0.717-1.205	0	Fixed	0.581
GG vs. AG+AA	Overall (fulfill HWE)	2	1291/397	0.783	0.601-1.021	0	Fixed	0.071
	Asian	2	1291/397	0.783	0.601-1.021	0	Fixed	0.071
	AgP	2	1291/397	0.783	0.601-1.021	0	Fixed	0.071
AG vs. AA	Overall (fulfill HWE)	2	1291/397	1.003	0.762-1.321	0	Fixed	0.983
	Asian	2	1291/397	1.003	0.762-1.321	0	Fixed	0.983
	CP	2	1291/397	1.003	0.762-1.321	0	Fixed	0.983
GG vs. AA	Overall (fulfill HWE)	2	1291/397	0.785	0.569-1.082	0	Fixed	0.140
	Asian	2	1291/397	0.785	0.569-1.082	0	Fixed	0.140
	CP	2	1291/397	0.785	0.569-1.082	0	Fixed	0.140

## Data Availability

All data generated or analyzed during this study are included in this article.
